# Room temperature processed protective layer for printed silver electrodes†

**DOI:** 10.1039/d3ra02212a

**Published:** 2023-07-10

**Authors:** Chungil Kim, Jin Ho Park, Jaehwan Ko, Suwoon Lee, Ri Gyeong Kwon, Subin Lee, Hangil Lee, Jun Young Kim, Hyung-Jun Song

**Affiliations:** a Department of Safety Engineering, Seoul National University of Science and Technology Seoul 01811 Korea hj.song@seoultech.ac.kr; b Department of Semiconductor Engineering, Gyeongsang National University Jinju 52828 Korea kimjy86@gnu.ac.kr

## Abstract

Low-temperature processed printed silver electrodes pave the way for electrical connections in flexible substrates with reduced energy consumption. Despite their excellent performance and simple process, printed silver electrodes' poor stability limits their applications. This study demonstrates a transparent protective layer without thermal annealing for printed silver electrodes, which maintains its electrical properties for a long period of time. A fluoropolymer, specifically a cyclic transparent optical polymer (CYTOP), was used as a protective layer for silver. The CYTOP is room temperature processable and chemically stable against carboxyl acid. The introduction of the CYTOP film on the printed silver electrodes mitigates the chemical reaction between silver and carboxyl acid, thereby elongating its lifetime. Under heated acetic acid, the printed silver electrodes with a CYTOP protective layer maintained their initial resistance for up to 300 hours, while the electrodes without a protective layer were damaged within a few hours. A microscopic image shows that the protective layer enables printed electrodes to maintain their shape without damage. Hence, the protective layer guarantees the accurate and reliable performance of electronic devices with printed electrodes under actual operating conditions. This research will contribute to designing chemically reliable flexible devices in the near future.

## Abstract 2

Room temperature processed CYTOP layer can effectively protect underlying printed silver electrodes from various kinds of chemicals, contributing to improved reliability of silver electrode.

## Graphical abstract

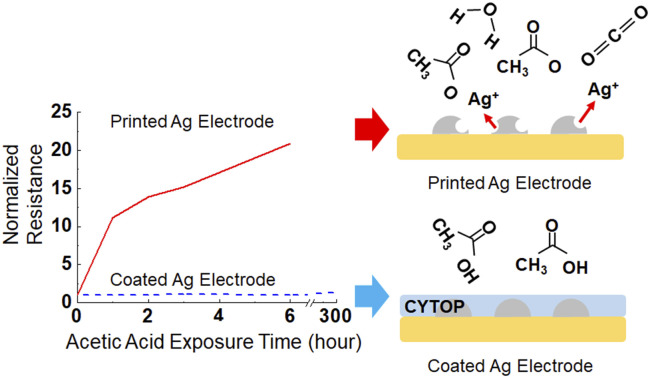

## Introduction

The printed electrode allows for the fabrication of electronic devices with reduced consumption of energy and materials by selectively depositing a film in a designated area. Moreover, these printed electrodes can remove complicated photo-lithography processes for electrode fabrication, thereby securing product price competitiveness in the market.^1–3^ The combination of a flexible substrate and printed electronics has expanded the application of electronic devices to wearables and curved designs. A low-temperature process is desirable for printed electrodes due to the low melting temperature of the flexible plastic substrate (∼150 °C). The advantage of low-temperature processed printed electrodes is that they enable electrode formation on various substrates while significantly lowering the process price. Various polymer and organic functional groups have been applied to metal particles for low-temperature processed electrodes, enhancing their dispersity in solution.^4,5^ The encapsulant polymer and function groups for a printed metal particle are removed during the post-annealing process. However, these are not wholly removed from the metal layer and negatively affect the printed electrodes' reliability. Moreover, poor adhesion between the substrate and printed electrode has been a challenge in low-temperature-processed films. As a result, the lifetime of low-temperature processed printed electrodes is typically shorter than those processed through film plating and physical vapor deposition.^6,7^ The poor stability of the printed electrodes is critical to the reliability of the entire system.

Thus, intensive studies have been conducted to improve the reliability of low-temperature processed electrodes from the perspective of mechanical stress, temperature tolerance,^8^ moisture,^9^ and chemicals. In particular, introducing a protective layer provides improved mechanical adhesion and a moisture barrier for the printed electrode. Graphene, graphene oxide, and thin metal oxide films have been adopted as protective layers for solution-processed electrodes.^10–14^ Although these thin protective layers enhance the printed electrodes environmental and mechanical stability without deteriorating their electrical function, the stability of printed electrodes against chemicals has not been studied intensively. Depending on the application, a device with printed electrodes may operate in a harsh environment with various chemicals and would encounter an acidic atmosphere due to the degradation of insulation and encapsulation materials. The oxidation of ethylene and vinyl compounds, widely used as encapsulation, and cable insulation materials, leads to the formation of carboxylic acid under high temperatures and intense light irradiation.^15^ As a result, the printed electrode may react with these materials during operation. Among the many printed electrode metal layers available, silver, whose resistivity is very low, is vulnerable to organic acid.^16–19^ The organic acid degrades the already poor mechanical adhesion of solution-processed silver electrodes after long-term exposure to heat and light. Consequently, the printed electrodes must be protected with a proper barrier film.

The preferred properties of the protective layer can be achieved through processing at low temperatures to accommodate the flexible substrates. Moreover, a transparent protective layer expands the application of printed electrodes to optoelectronic devices. A metal oxide-based transparent layer is a candidate for protecting printed electrodes from chemicals and moisture; however, the brittle metal oxide layer can crack after stretching the substrate several times. Additionally, the high processing temperature might damage the underlying printed electrode. Alternatively, a low-temperature processable polymer-based protective layer is more flexible than an inorganic film.^20,21^ Nevertheless, the polymer layer's poor chemical resistance and water-repellent features need to be improved. Considering the pros and cons of polymer and metal oxide films, a multi-stack hybrid protective layer has been adopted in previous works.^22–24^ However, hybrid protective layers require a complicated process. Another possible solution for the protective layer is reliable polymer resin, which is widely adopted in the semiconductor industry. However, these layers require a high-temperature annealing process and strong UV irradiation, which might threaten the substrate, semiconducting polymer, and silver electrodes.^25,26^ As a result, a new type of low-temperature and simply processed polymer protective layer, stable to chemicals, will be more beneficial for improving the reliability of devices with printed electrodes.

Hence, this study demonstrates reliable printed electrodes by the implementation of a fluoropolymer coating. A fluoropolymer, namely a cyclic transparent optical polymer (CYTOP), is a room temperature processable, and chemically stable layer without any requirement for thermal annealing. An inkjet-printed silver electrode protected by a CYTOP maintained its conductivity for up to two weeks under acetic acid, while an electrode without the protective layer was damaged by acetic acid within one hour at high temperatures. The statistical analysis and microscopic images indicate that the protective layer prevented silver electrodes from detaching from the substrate. In addition, the protective layer was adequate in mitigating the chemical reaction between the acetic acid and the silver electrode. Therefore, the CYTOP protective layer makes printed electrodes more reliable and safer. Thus, the results shown here will become a guideline for developing a chemically stable printed electrode.

### Experiments

**Fig. 1:**
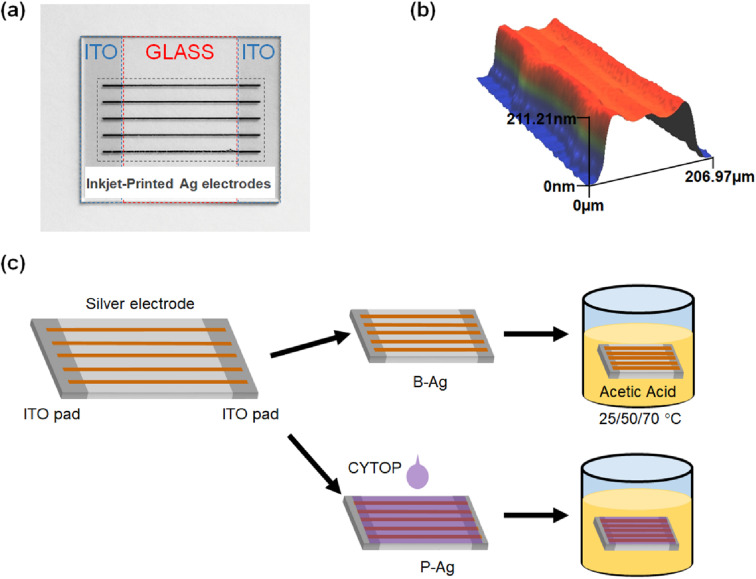
(a) Photo of the silver electrode printed on the ITO-patterned glass. The silver electrode electrically connects two separate ITO contact pads. The resistance between the two ITO pads increases as the silver electrode is damaged. (b) 3D profile image of the inkjet printed electrode as fabricated. (c) Schematic of the silver electrode reliability experiment against acetic acid. The silver electrode with a protective layer (P-Ag) was compared to the electrode without one (B-Ag). Here, the silver electrode-printed glasses were immersed in acetic acid under various surrounding temperatures.

To quantify the effect of acetic acid on the performance of the electrodes, inkjet-printed silver electrodes were formed on indium tin oxide (ITO) patterned clean glass (25 × 20 mm^2^), as shown in Fig. 1a. The silver electrodes were formed using silver ink (TEC-IJ-060 from InkTec) through an inkjet printer (DMP-2831, Fujifilm Dimatix). The silver electrodes horizontally connect two separated ITO contact pads on both glass edges. Here, the silver electrodes were annealed at 150 °C for one hour and had a width and height of 201.71 μm and 202.01 nm, respectively (Fig. 1b). To protect the silver electrode from chemicals, a transparent, solution-processed, protective fluoropolymer, CYTOP (Asahi glass), was spin-coated on the silver electrode-printed glass (1000 rpm, 30 seconds).^27–30^ CYTOP has a high carbon–fluorine binding energy, so it is ordinarily robust to harsh outdoor conditions, such as heat, UV light, and moisture.^31–36^ Hence, it has been widely used as an encapsulation material for thin film devices. After the protective layer was cast on the glass, it was dried in ambient conditions (25 °C) for 30 minutes. Since the film does not require any annealing under high temperatures or light irradiation, it is quite suitable for flexible electrodes. Finally, the silver electrodes with, and without protective layers were immersed in acetic acid, as shown in Fig. 1c. The oxidation of ethylene and vinyl compounds (widely used for encapsulation and cable insulation materials) led to carboxylic acid formation.^37^ Hence, acetic acid was chosen to evaluate the effect of the CYTOP protective layer on the underlying silver electrode. Hereafter, the silver electrode with a CYTOP layer was termed P-Ag, while the bare electrode without a protective layer was termed B-Ag. In addition, the experiments were conducted at 25 °C, 50 °C, and 70 °C to determine the effect of surrounding temperature on the degradation of low-temperature processed silver electrodes.

The resistance between two ITO contact pads was repeatedly monitored to evaluate the status of the silver electrodes using a digital multimeter (DMM6500, Keithley) with a probe station. In addition, the microstructure of the silver electrodes was obtained by an optical microscope (BX-53F2, Olympus) and an optical 3D profiler (NV-1800, Nano System).

## Results and discussion

**Fig. 2:**
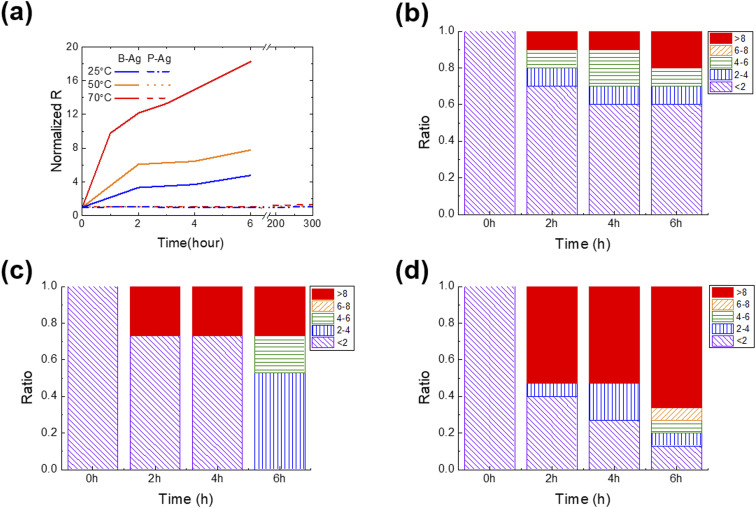
(a) Normalized average resistance of silver electrodes with and without the CYTOP protective layer immersed in acetic acid. The CYTOP layer protects the silver layer from acetic acid and increases its lifetime. Statistical analysis of the resistance of B-Ag stored at (b) 25 °C, (c) 50 °C, and (d) 70 °C. The change of resistance is classified into five subgroups compared to its initial value.

Fig. 2 shows the change in normalized resistance (NR) of B-Ag and P-Ag between two separated contact pads (ITO) obtained by an average of 10 samples for each case. Here, the NR is derived by dividing the resistance between pads after exposure to acetic acid by the initial resistance. As shown in Fig. 2, acetic acid exposure significantly increased the NR of B-Ag. For example, the NR of B-Ag increased by a factor of four within six hours at 25 °C. As the surrounding temperature elevated, the chemical reaction between acetic acid and the silver electrode was facilitated, and the electrode decayed faster. Thus, the NR of B-Ag increased by more than a factor of six within two hours at 50 °C. As soon as it was exposed to acetic acid at 70 °C, the NR rapidly increased. The resistance increased by a factor of 11 in less than one hour at 70 °C. Hence, it is apparent that an inkjet-printed silver electrode is susceptible to organic acid and decays much faster under high temperatures.

Introducing the CYTOP protective layer to the silver electrode makes it more robust to acetic acid. The P-Ag maintained its resistance despite long-term exposure to acetic acid. At any temperature, the thin CYTOP film (∼1 μm) protected the electrode from the acid. For instance, the resistance of P-Ag was similar to its initial value after over 300 hours of exposure to acetic acid at a high temperature (70 °C). If the acetic acid contacts the silver electrode, the electrode will decay, and the reaction speed is proportional to the surrounding temperature. However, the P-Ag kept its initial resistance independent of the surrounding condition. This implies that the CYTOP mitigates the chemical reaction between silver electrodes and acetic acid by physically separating them. Moreover, the contact angle of acetic acid on the CYTOP layer is much larger than that on a glass substrate, resulting in poor penetration of acetic acid in silver electrodes with a CYTOP layer (please see Fig. S2† for more details). Although pores were located in the CYTOP film due to the unoptimized coating process, the penetrated acetic acid had a lower chance of spreading on the glass surface due to high surface energy. Therefore, the printed silver electrode with a CYTOP layer is more reliable when exposed to organic acid.

As discussed earlier, the chemical reaction between the silver electrode and acetic acid strongly affects its resistance. If the acid degrades the surface of the silver electrode, its resistance value will gradually increase. However, the resistance value will suddenly rise if the weakly bound printed silver film detaches from the substrate or neighboring electrode. To determine the underlying mechanism and which part of the electrode was corroded by acetic acid, the statistical change of resistance was analyzed as a function of exposure time under various surrounding temperatures (Fig. 2b–d). P-Ag did not exhibit a significant resistance increase that was dependent on the exposure time (not shown in the figures). All P-Ag samples kept their initial resistance after acetic acid exposure. This indicates that the CYTOP protective layer does protect not only the surface of the printed silver electrode but also the interface between the silver electrode and the glass substrate.

Interestingly, the primary degradation mechanism within NR differs depending on the exposure time and temperature. Acetic acid destroys a part of the weakly bound B-Ag, as soon as there is contact. Under all tested cases, the B-Ags exhibited a sudden increase in NR (>eight times their initial value) within two hours of exposure to acetic acid. However, this phenomenon more frequently occurs at high temperatures. The rapid increase in resistance is mainly due to electrode detachment from the substrate. Hence, it is plausible that the chemical reacts very actively with B-Ag, and its weak part is detached from the neighboring electrode and substrate at the beginning stage of acetic acid exposure. After two hours of exposure to acetic acid, the resistance of B-Ag gradually increased, indicating that the chemical reaction on the surface of the silver electrodes mainly degrades the electrical connection. All B-Ag cases showed a similar degradation trend independent of the temperature. However, ratio of a sudden resistance increase was more dominant at higher temperatures due to the increased chance of chemical reaction.

**Fig. 3:**
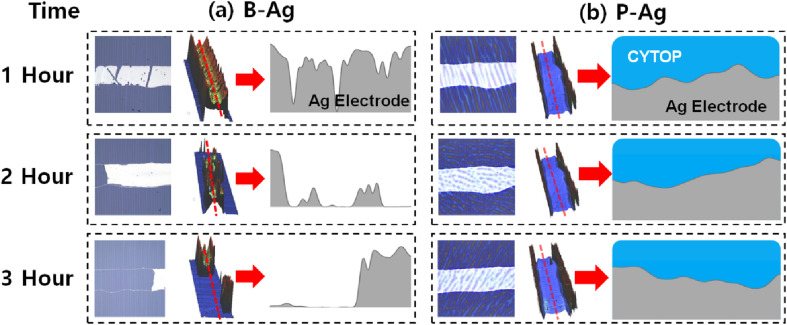
Microscopy image (left) and 3D optical profile (middle) of (a) B-Ag and (b) P-Ag under acetic acid exposure at 70 °C. The printed silver electrode's corresponding cross-sectional images (marked as the red line in the middle image) are also displayed on the right (obtained from different samples, not the same sample). Here, the 3D optical profile detects the topology of the film using the reflectance difference of the underlying layers. Since the CYTOP is optically transparent, the 3D optical profiler only detects the topology of the printed silver electrode in the P-Ag. In contrast, the morphology of the CYTOP layer is mainly measured by the 3D optical profiler, where the silver layer does not exist in the P-Ag. It resembles a wall around the silver electrode in the image. The part of (a) B-Ag is broken by acetic acid, while the (b) P-Ag keeps its shape regardless of acetic acid exposure. The microstructure analysis also indicates that introducing CYTOP to the printed electrode makes it reliable against acetic acid. The image after three hours of exposure is shown in Fig. S2 and S3.†

The topology changes of B-Ag and P-Ag are consistent with their resistance change under acetic acid at 70 °C. Fig. 3, S2 and S3† show the microstructures of B-Ag and P-Ag depending on the acetic acid exposure time. Significant damage was not observed in the P-Ag (please see Fig. S2 and S3†). The P-Ag kept its initial microstructure despite acetic acid exposure, which is similar to the unchanged NR of P-Ag. The P-Ag is continuously connected despite acetic acid exposure, as shown in their cross-sectional image. The P-Ag is continuously connected despite acetic acid exposure, as shown in their cross-sectional image. In contrast, the B-Ag began to degrade after one hour of acetic acid exposure. Part of B-Ag detached from the substrate due to the chemical reaction between B-Ag and acetic acid. As the exposure time increased, the portion of the detached silver electrodes enlarged in the B-Ag. The CYTOP is also effective in protecting the electrodes from a solution with chlorite, which might reveal its versatile performance for keeping the electrodes from chemicals (please see Fig. S4†). The NR and microstructure analysis of printed silver electrodes indicates that the CYTOP effectively protects the underlying electrode, guaranteeing the printed electrode's reliability.

A polymer, whose mechanical and chemical properties are similar to those of CYTOP, would be a candidate for protecting silver electrodes from an organic acid by physically separating the electrode from the acid. To find alternatives to CYTOP, other room-temperature processable and chemically stable polymers, acryl resin and perhydropolysilazane, were applied to silver electrodes to protect the layers from an organic acid.^38,39^ However, their poor adhesion to glass and non-uniform film formation hampers their application as alternative protective layers for silver electrodes (please see Fig. S4 and S5†). Thus, the CYTOP's unique features, reliable performance under acid, good adhesion to glass, and uniform film formation make it a suitable protective layer for printed electrodes.

**Fig. 4:**
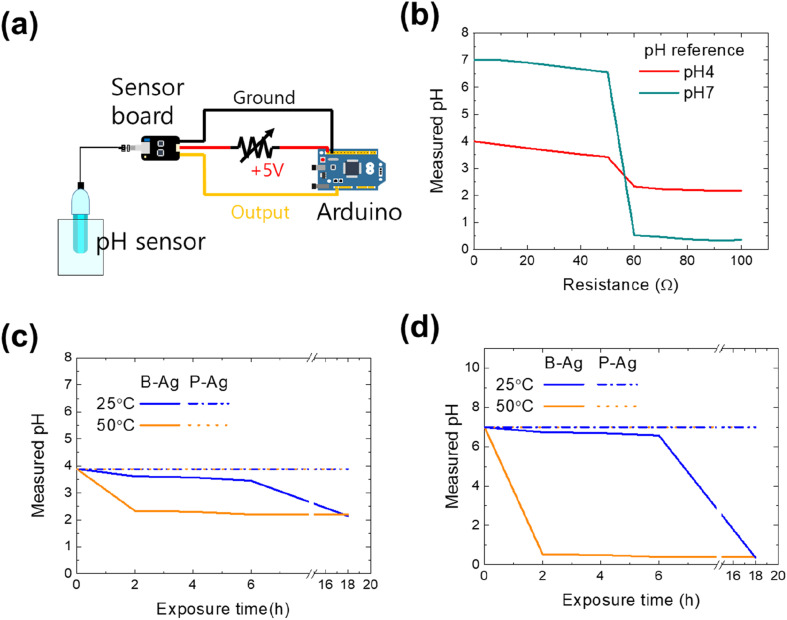
(a) Schematic of the pH meter connected with the Arduino. Here, the printed silver electrode was assumed to be applied to the power cable between the sensor board and Arduino. If the cable is exposed to acetic acid, the resistance of the cable will increase. (b) The measured pH of two standard buffer solutions (pH 4 and 7) *versus* the resistance of the electrical cable. As the resistance of the output cable increased, the measured pH value decreased in the sensor. The measured pH of (c) pH 4 and (d) pH 7 standard buffer solutions with the electrical cable, including B-Ag, and P-Ag. Because of the increased resistance of B-Ag under acetic acid exposure, the sensor does not accurately monitor the pH value of solutions after exposure to acetic acid.

As aforementioned, the printed silver electrode is vulnerable to acetic acid, while the electrode with the CYTOP protective layer maintains its performance. The reliability of the printed electrodes would affect the entire system that comprises them. Thus, we examined how the chemical reliability of printed electrodes affects the whole system. The first set of experiments used a pH monitoring sensor to control the chemical process or detect specific materials. Since the pH monitoring system aims to determine a solution's accurate pH value, the system will frequently operate in an environment of acid solution and vapor. Fig. 4 shows the schematic of the pH measurement system consisting of an Arduino (UNO R3), a sensor board, and a pH sensor (SEN0161, DFRobot), whose measuring range is pH 0–14. The sensor's output voltage varies depending on the hydrogen ion concentration of the solution in normal operation, thereby detecting the exact pH value of a solution. However, its value would be inaccurate if the resistance of the power cable increased due to exposure to acetic acid (please see Fig. 4b). Using two standard buffer solutions (pH 4 and 7, Samchun Chemicals) and a variable resistor (0–100 Ω), the effect of a printed silver electrode's increased resistance on the pH monitoring system was quantified. The measured values of the standard solutions were the same as those provided by the manufacturer when the resistance was below 50 Ω. However, the values dropped sharply when the resistance was over 50 Ω. The reason for incorrect measurements with high resistance in the power cable is the power not being correctly provided to the amplifier and measurement system due to ohmic losses. Thus, the system is expected to detect inaccurate values if the printed electrode is exposed to acid.

Fig. 4c and d are the pH value of buffer solutions through the pH monitoring system with P-Ag and B-Ag as a function of acetic acid exposure time. The experiments were conducted using two different standard buffer solutions (pH 4 and 7). Here, it was assumed that the P-Ag and B-Ag were a part of the power cable and were continuously exposed to the acetic acid. In addition, their resistance value affected the measured result, based on Fig. 4b. The system with a proper protective layer (P-Ag) detected accurate pH values of standard buffer solutions despite acetic acid exposure. In contrast, the corrosion of B-Ag caused measurement errors within 18 hours of acetic acid exposure at 25 °C. The degradation of the pH monitoring system was more dominant at high temperatures. The system became useless within two hours of acetic acid exposure at 50 °C. Such a significant drop in the measured value made the monitoring system unstable and unreliable. Hence, applying the suggested protective layer to the printed silver electrode is very beneficial for precise and accurate measurement.

**Fig. 5:**
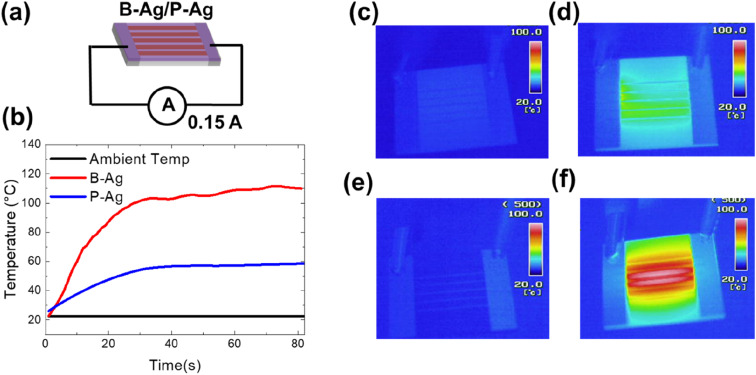
(a) Schematic of the joule heating experiment with a degraded printed electrode, where 0.15 A of current continuously flows through the electrode. In contrast, the electrode temperature is monitored by a thermal imaging camera and K-type thermocouple. (b) Temperature and current flow of B-Ag and P-Ag exposed to acetic acid for six hours at room temperature. Thermal camera images of (c, d) P-Ag and (e, f) B-Ag initially and after one minute of current flow, respectively.

In addition, an increased resistance of the printed silver electrode may not only affect the system's performance but may also increase the risk of fire. The increased fire risk is attributed to joule heating by the degraded silver electrode. Fig. 5 shows the temperature of B-Ag and P-Ag exposed to acetic acid for six hours at 25 °C, where 0.15 A of current continuously flows. In the case of B-Ag with corroded silver electrodes (∼110 Ω), the temperature of the electrode increased by more than 100 °C within one minute. The heating mainly occurred at the damaged silver electrode, as shown in the thermal image of B-Ag (Fig. 5e and f). The heating caused by the damaged electrode can burn devices or melt the installation layer and plastic substrate. Moreover, the B-Ag became hot enough to ignite combustible materials. In contrast, the temperature of P-Ag was only elevated to 60 °C because of its low resistance (∼20 Ω). The thermal images of P-Ag indicate that the protective layer keeps the silver electrode from overheating by blocking its corrosion under acetic acid (please see Fig. 5). Consequently, it is apparent that the CYTOP protective layer provides stable performance and reduced risk to the system containing a printed silver layer.

## Conclusion

In this study, the effect of a CYTOP protective layer on the reliability of solution-processed silver electrodes was determined. The acetic acid exposure experiment indicated that the CYTOP layer protected the underlying silver electrode from chemicals while the unprotected silver electrode actively reacted with acetic acid. The resistance of B-Ag increased tenfold within two hours at a higher temperature. The P-Ag, however, maintained its initial resistance for more than two weeks regardless of the surrounding temperature, revealing that the protective layer effectively mitigates chemical reactions between silver electrodes. As the stability of the silver electrode is improved by introducing the protective layer, the system equipped with the same would operate accurately for longer while reducing power loss due to joule heating. Therefore, the CYTOP protective layer contributes to improving the reliability of a system containing printed silver electrode.

## Conflicts of interest

There are no conflicts to declare.

## Supplementary Material

RA-013-D3RA02212A-s001
