# Supplementary material for: Room temperature processed protective layer for printed silver electrodes
Source: RSC Adv. 2023 Jul 10;13(30):20557–64. doi: 10.1039/d3ra02212a (PMC10331791; doi:10.1039/d3ra02212a)
Supplement: RA-013-D3RA02212A-s001 [file RA-013-D3RA02212A-s001.pdf]

Electronic Supplementary Material (ESI) for RSC Advances.
This journal is © The Royal Society of Chemistry 2023

[Supplementary information]

Solution processed protective layer for printed silver electrode against Acetic acid

Chungil Kim[1,#], Jin Ho Park[2,#], Jaehwan Ko[1], Suwoon Lee[1], Ri Gyeong Kwon[2], Subin Lee[1], Hangil Lee[1], Jun Young Kim[2], Hyung-Jun Song[1]

1. Department of Safety Engineering, Seoul National University of Science and Technology, Seoul, 01811, Korea
2. Department of Semiconductor Engineering, Gyeongsang National University, Jinju, 52828, Korea

Corresponding authors: J.Y. Kim (kimjy86@gnu.ac.kr), H.-J Song (hj.song@seoultech.ac.kr)

[#] These authors equally contributed this work.

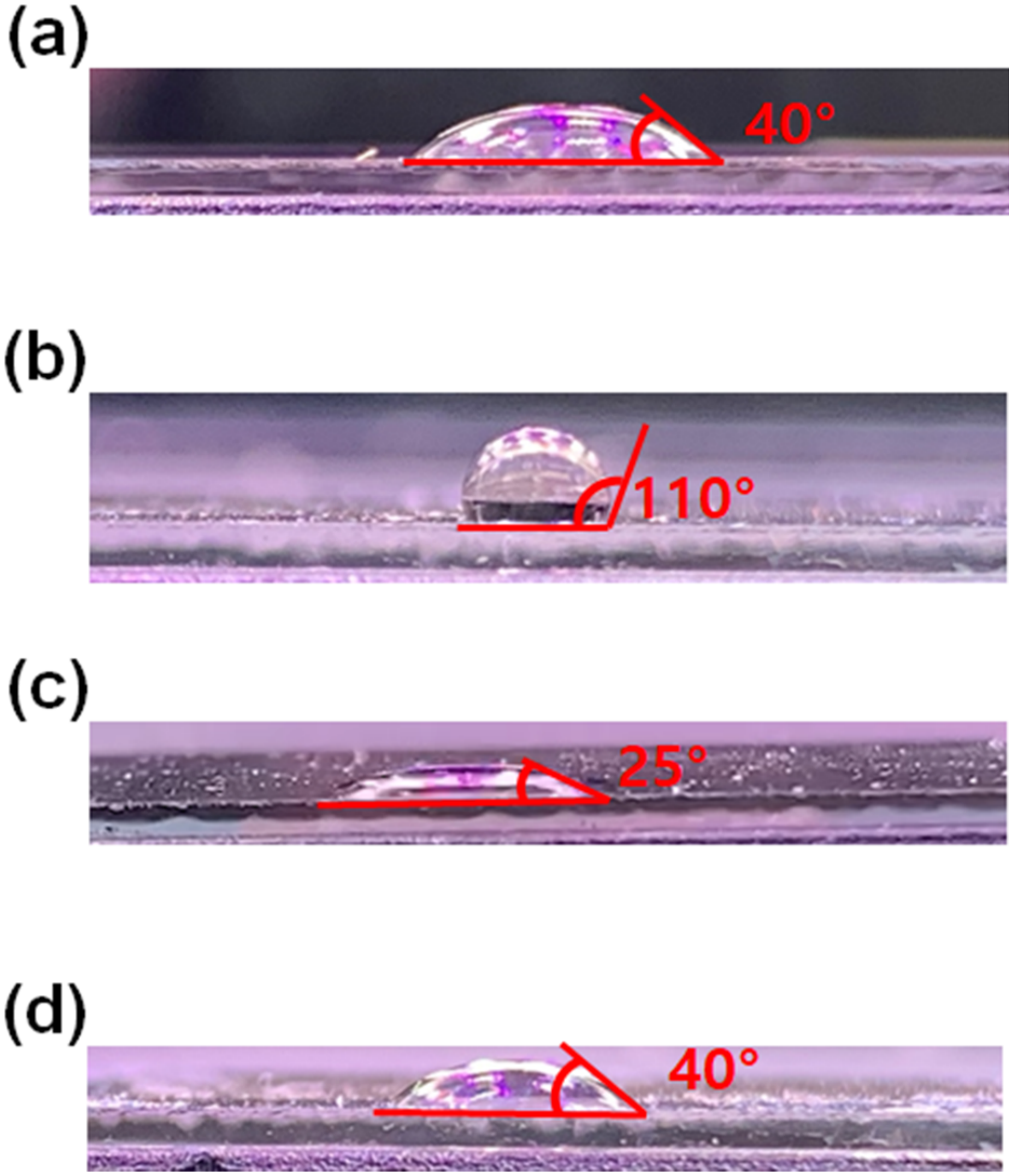

Figure S1. Contact angle for water and acetic acid on (a,c) the bare glass and (b,d) the CYTOP film, respectively

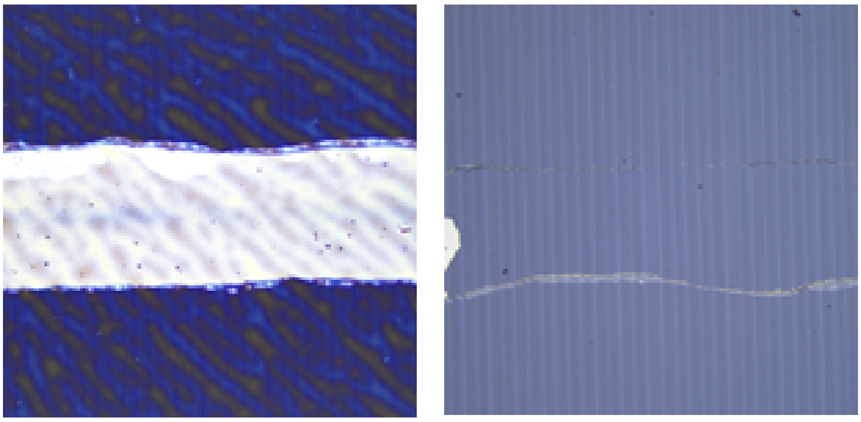

Figure S2. Microscopy of silver electrode corrosion after 4 hours of acetic acid exposure. The left of the image is the P-Ag, while the right of image is B-Ag.

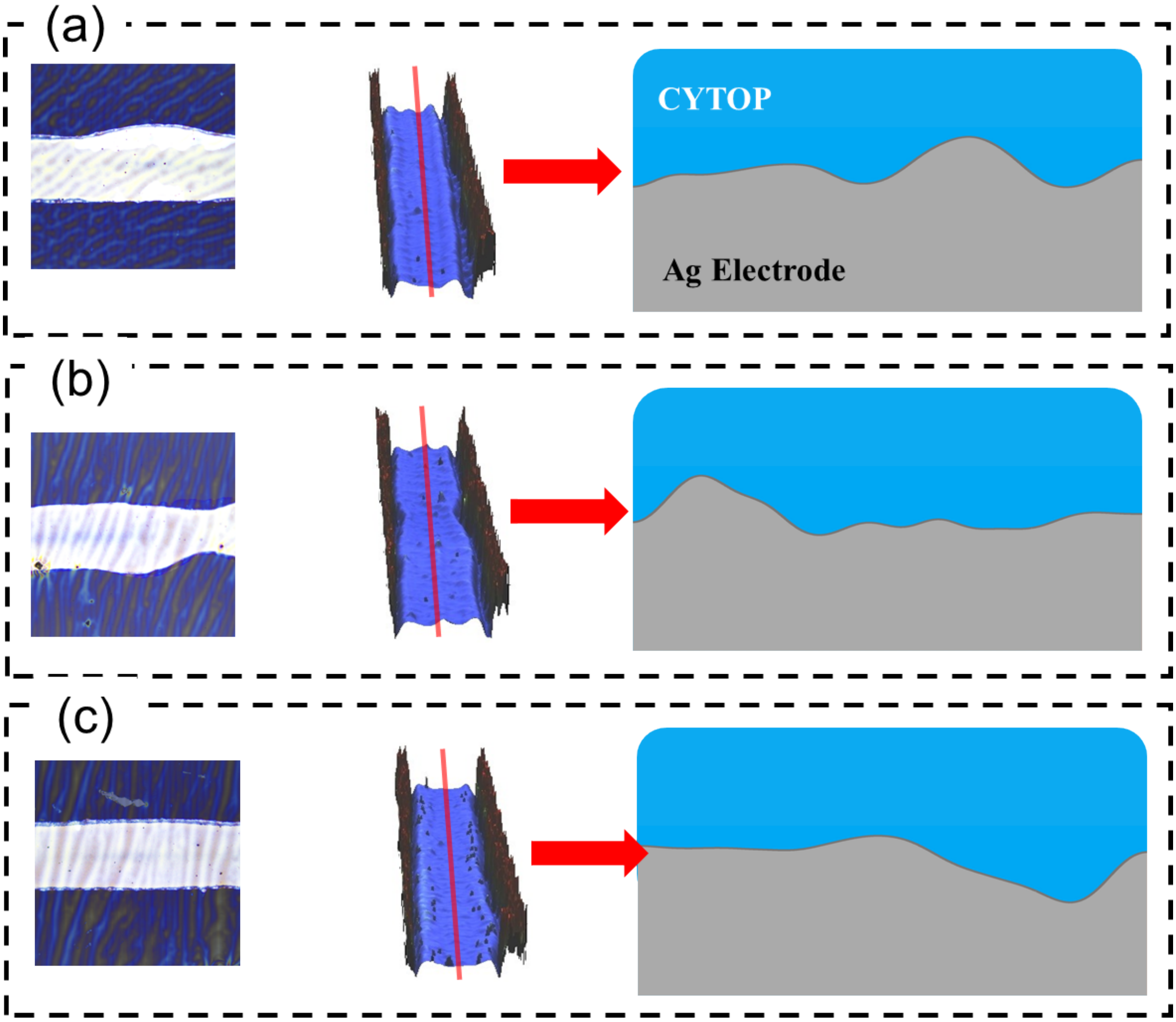

Figure S3. Microscopy image (Left), 3D optical profile (Middle), and corresponding cross-sectional image (Right) of P-Ag under heated acetic acid (70 °C) for (a) 5, (b) 6, and (c) 7 hours.

Protective properties of CYTOP against other chemicals

The desirable property of a protective layer is to keep electrodes from various types of chemicals. To validate the CYTOP protective layer's versatility, we monitored the silver electrode with and without a protective layer under sodium hypochlorite solution. Usually, silver quickly reacts with sodium hypochlorite and changes into silver hypochlorite [1,2]. The chemical reaction formula is as follows.

$$NaClO(aq) + Ag \rightarrow AgClO(aq) + Na^{+}$$

Thus the silver layer without a protective layer disappeared within 1 hour of exposure to sodium hypochlorite solution, as shown in Figure S4. However, the electrode with CYTOP is well preserved under sodium hypochlorite solution, which indicates that the CYTOP protective layer is versatile for various kinds of solutions.

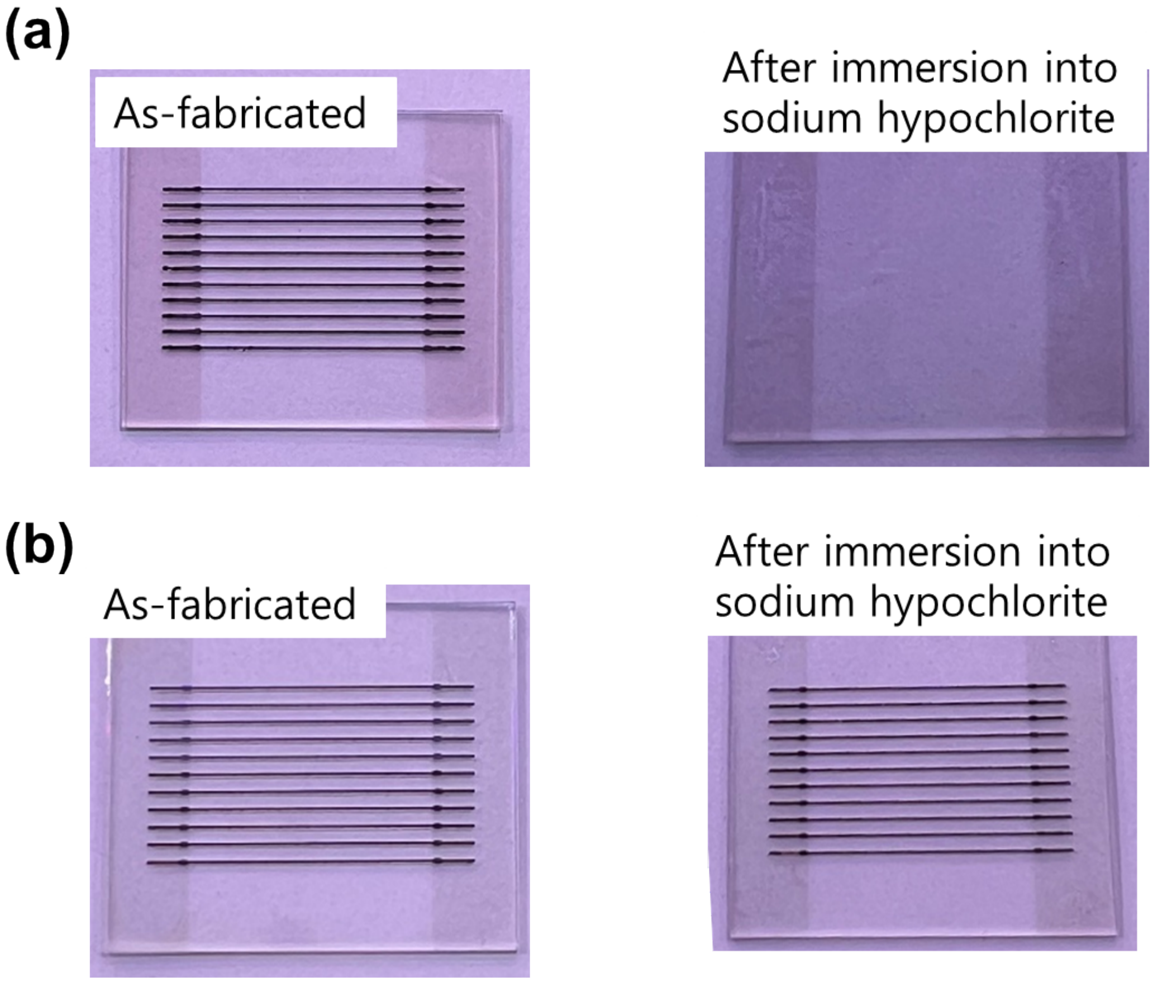

Figure S4. Photographs of (a) the B-Ag and (b) the P-Ag under sodium hypochlorite solution (NaClO in water). Under sodium hypochlorite solution, the silver electrode without a protective layer is fully corroded within 1 hour. On the other hand, the CYTOP effectively hinders the chemical reaction between silver and sodium hypochlorite.

Effect of other protective layers on the stability of silver electrode

We compared the effectiveness of the CYTOP with other low-temperature processable polymer layers. One is perhydropolysilazane, widely used for water and chemical-proof film [3]. And the other is an acrylic resin used for a protective layer of printed circuit boards [4-5]. The electrodes with both protective layers show a rapid resistance value increase after immersion into acetic acid, as shown in Figure S5. Due to poor adhesion between the substrate and protective layer, some of the electrodes are detached from the substrate after acetic acid exposure (See Figure S6). Hence, the room temperature processable protective layer should be carefully chosen its chemical resistance as well as adhesive properties to the substrate.

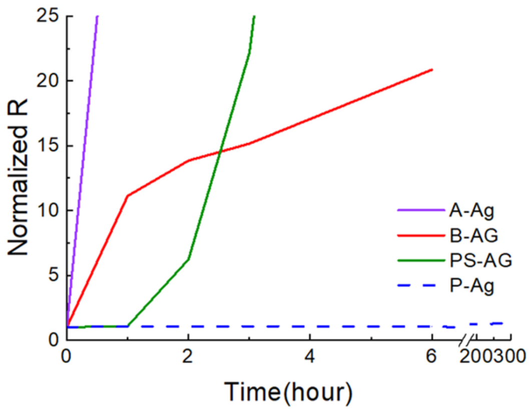

Figure S5. Normalized resistance- chemical exposure time characteristics of the silver electrode with various protective layers under high-temperature acetic acid (70 °C). Three different coating materials, acrylic resin (A-Ag), perhydropolysilazane film (PS-Ag), and CYTOP film (P-Ag), were used in this experiment.

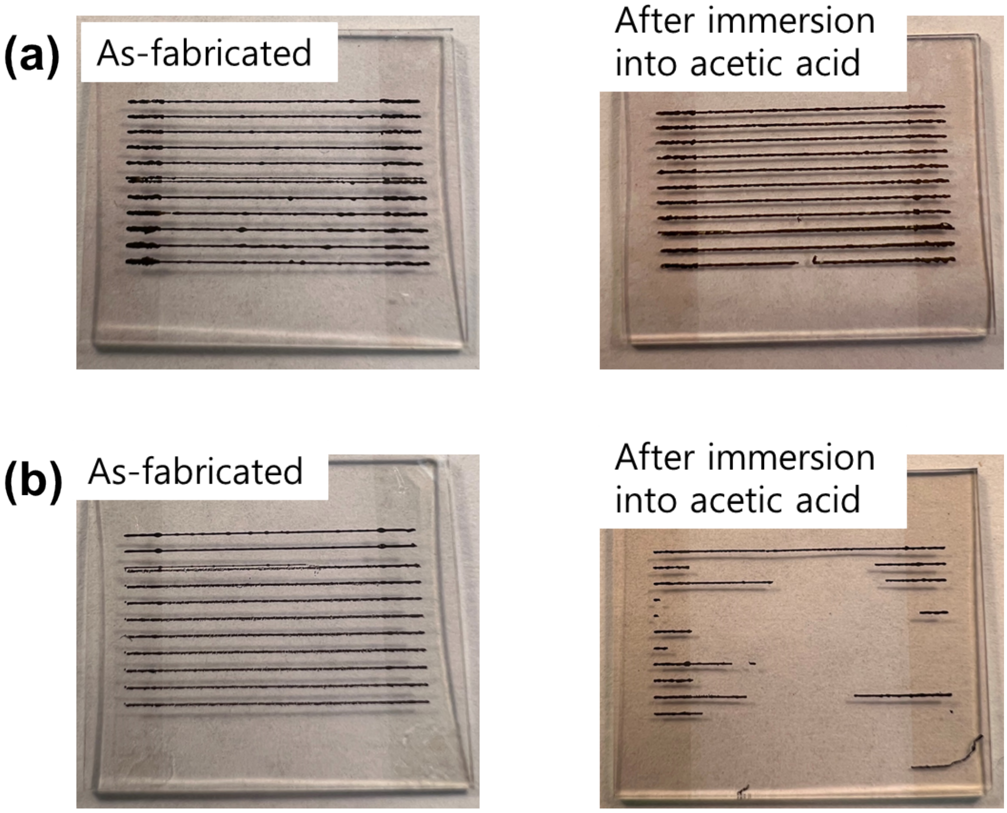

Figure S6. Photographs of silver electrodes with (a) perhydropolysilazane protective film and (b) acrylic resin. After immersion in acetic acid, the silver electrode is damaged due to poor adhesion of the protective layers to a glass. The image was taken after 4 hours of acetic acid in the polysilazane protective layer. On the other hand, the image of the electrode was obtained after 30 minutes of exposure to acetic acid in the case of the acryl-based protective layer.

References

[1] Z. Zhang, H Guo, C Ma, M Xia, J. C. White, B. Xing, L. He, Rapid and efficient removal of silver nanoparticles from plant surfaces using sodium hypochlorite and ammonium hydroxide solution, Food Control, 98, 68-73 (2019)

[2] G. V. K. Puvvada, D.S.R. Murthy, Selective precious metals leaching from a chalcopyrite concentrate using chloride/hypochlorite media, Hydrometallurgy, 58, 185-191 (2000)

[3] L. Sun, K. Uemura, T. Takahashi, T. Yoshida, and Y. Suzuri, Interfacial Engineering in Solution Processing of Silicon-Based Hybrid Multilayer for High Performance Thin Film Encapsulation, ACS Applied Materials & Interfaces 11 , 43425-45432, 2019

[4] L. Shen, Y. Li, J. Zheng, M. Lu, K. Wu, Modified epoxy acrylate resin for photocurable temporary protective coatings, Progress in Organic Coatings, 89, 17-25, 2015

[5] U. Rathinavelu, M. S. Jellesen; P. Moller; R. Ambat, Effect of No-Clean Flux Residues on the Performance of Acrylic Conformal Coating in Aggressive Environments, IEEE Transactions on Components, Packaging and Manufacturing Technology, 2, 179, 2012
